# Take the Right Turn: The Role of Social Signals and Action–Reaction Sequences in Enacting Turning Points in Negotiations

**DOI:** 10.1007/s10726-020-09664-4

**Published:** 2020-03-18

**Authors:** Michele Griessmair, Johannes Gettinger

**Affiliations:** 1 Visiting Research Fellow, Sir Zelman Cowen Centre, Victoria University, Melbourne, Australia; 2 Faculty of Business, Economics and Statistics, University of Vienna, Oskar-Morgenstern-Platz 1, 1090 Vienna, Austria; 3 Institute of Interorganisational Management and Performance, University of Hohenheim, Stuttgart, Germany

**Keywords:** Negotiation, Turning points, Social signals, Emotions, Interacts

## Abstract

Negotiations and conflicts do not evolve smoothly but are discontinuous involving transitions, break-, and turning points that change the flow of the negotiation. Given that these departures may be decisive in determining whether the involved parties come to a successful conclusion, several scholars have pointed out the importance of investigating whether impasse and settlement dyads exhibit different turning point profiles. To address this question, we extended Druckman’s (J Confl Resolut 45:519–544, 2001) turning point model by integrating interlocking action–reaction sequences that initiate and (dis)confirm the departure from zero-sum bargaining. Furthermore, we consider social signals as previously not addressed class of events triggering the turning point. We propose and show that social signals act as precipitants to substantive change at the offer level and that how negotiators enact the action–reaction sequences discriminates between successful and unsuccessful dyads.

## Introduction

Negotiations are an inevitable and ubiquitous part of organizational life which take place not only during business deals between organizations but also when coordinating activities within the organization to meet organizational goals (Olekalns and Weingart 2003, 2008). Failed negotiations and unresolved conflicts “can result in missed opportunities and large financial losses and can threaten the very survival of organizations” (Olekalns and Weingart 2008: 135). Yet, they are common within and between organizations, costing the involved parties a significant amount of time, energy, and money (Brett et al. 1998). The detrimental impact of ongoing conflicts and failed negotiations are described in numerous examples (see, for instance, Arrow et al. 1995; Brett et al. 1998).

Despite being aware of the destructive consequences, parties often appear unable to turn the situation around (Brett et al. 2004). Consequently, research has devoted considerable effort to investigating the dynamics bringing negotiators closer or farther away from an agreement (Adair and Brett 2005; Olekalns and Weingart 2003; Weingart and Olekalns 2004). A common theme in this literature is that negotiations do not evolve smoothly but are discontinuous (Druckman et al. 2009) and involve transitions (Olekalns et al. 2003), breakpoints (Brett et al. 2004), critical moments (Donohue 2004), and turning points (Druckman 2001)—observable events that change the flow of the negotiation (Donohue 2004).

The vast majority of previous research has considered turning points in retrospective case analyses of (international) conflicts or, when focusing on deal-making negotiations, in conjunction with (episodic) phase models (for reviews, see, Druckman 2004; Druckman 2017; Druckman and Olekalns 2013b). Phase models describe the negotiation process as unfolding series of stable episodes of coherent behavior, each having specific characteristics (Holmes 1992). Empirical studies in this tradition have not only demonstrated that negotiations resemble evolutionary rather than static processes but also that the timing with which certain negotiation behavior is shown differs between successful and unsuccessful negotiation dyads (Olekalns et al. 2003, 1996). This stream of research has focused on the behavior negotiators exhibit before and after the transition but does not specifically address how the change is initiated, what happens at the turning point, and which subsequent reactions direct negotiators closer or farther away from a settlement. Given that turning points may be decisive in determining whether the involved parties come to a successful conclusion, several scholars have pointed out the importance of addressing these questions (Donohue 2004; Druckman 2001; Druckman and Olekalns 2011, 2013b). However, as noted by Brett et al. (2004), only a limited amount of negotiation research specifically focuses on turning points.

The aim of this study is to address theoretically and empirically whether impasse and settlement dyads exhibit different turning point profiles with regard to substantive (offers and concessions) as well as social (expressed emotions) behavior. Using negotiation process analyses (e.g., Brett et al. 1998; Olekalns and Smith 2000; Olekalns et al. 1996; Weingart et al. 1999; Weingart et al. 2004), we conduct ex-post analyses of negotiation interactions examining negotiators’ behavior before and after the turning point that marks the transition from zero-sum bargaining towards exchanging fair offers and creating value. For this purpose, we extend Druckman’s (2001) turning point model by integrating interlocking action–reaction sequences that initiate and (dis)confirm the departure from zero-sum bargaining. Furthermore, we consider social signals as previously unaddressed events triggering the turning point. As will be discussed in more detail in the next section, we propose and show that social signals act as precipitants to substantive change at the offer level and that how negotiators enact the action–reaction sequences discriminates between successful and unsuccessful dyads.

## Theoretical Background

**Fig. 1:**
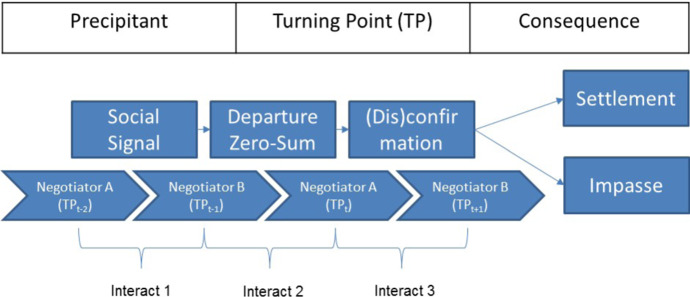
Theoretical framework of turning points and interacts

Turning points have been conceptualized differently in negotiation literature (Brett et al. 2004). In conjunction with episodic phase models, turning points are shifts from one phase to the next (Druckman 2001). The phases are stable episodes that are characterized by the uninterrupted use of tactics belonging to the same group with both negotiators predominantly following the same strategic orientation (Olekalns et al. 2003; Olekalns and Weingart 2008) or the same type of activity (Vetschera 2013). These periods of stability are interrupted when divergent strategic approaches and discrepant goals are prevalent and negotiators attempt to change the dynamics of the negotiation (Brett et al. 1998; Druckman and Olekalns 2011; Olekalns and Weingart 2008). Thus, within the episodic phase model approach the turning points mark the transition from one coherent set of activity to another or the change in strategic orientation by the negotiators. Other negotiation scholars have investigated turning points as “critical moments” or “critical incidents”—rarely occurring events that interrupt the negotiation process and direct it towards or farther away from a joint solution (Brett et al. 2004; Donohue 2004). In this view, turning points are salient incidents within the negotiation that change its course by causing negotiators to alter their strategy, the perception of and relationship with the counterpart, or the negotiation outcome (Druckman et al. 2009; Olekalns and Smith 2005). For the present study we employ the turning point framework developed by Druckman (2001) (see Fig. 1).

Druckman’s (2001) model provides a comprehensive characterization of turning points on a process as well as on a content level.^1^ On a process level, it first proposes three stages, each of which is instrumental in bringing negotiators closer or farther away from an agreement. Accordingly, (1) internal or external precipitants initiate (2) a clear departure from earlier patterns of the negotiation process that (3) leads to a consequence (Druckman 2001) (see Fig. 1). Furthermore, on a content level, Druckman’s turning point model provides a typology of potential precipitants and events that constitute departures. We extend Druckman’s model on a content level by introducing social signals as a new class of precipitants and on a process level by incorporating interacts (action–reaction sequences) in order to capture the interactive nature of the turning point stages.

Precipitants are “identifiable events that trigger changes in the negotiation process” (Druckman et al. 2009: 15). Extant research and typologies focus on substantive and procedural precipitants such as reframing issues or new ideas brought to the negotiation table (e.g., Druckman 2001; Druckman and Olekalns 2011). In our model, we investigate social signals as a new class of precipitants. Accordingly, the precipitants are constituted by changes in the emotional climate of the negotiation—we hypothesize that these destabilize the ongoing negotiation pattern and send a social signal to the counterpart indicating that a redirection of the negotiation is required.

When negotiators react to the precipitant, the negotiation process changes resulting in the turning point or departure (Druckman et al. 2009), the second step in Druckman’s (2001) model. Departures are clear and self-evident changes from earlier events or patterns (Druckman and Olekalns 2013b) such as procedural changes, incorporation of new ideas, or abandoning a give-and-take pattern (Druckman 2001). Following Griessmair and Druckman (2018), in the present study we conceptualize the turning point as a departure from zero-sum bargaining. Departures eventually lead to consequences, the final step in Druckman’s (2001) model. Consequences are defined as a “clear and self-evident impact of a departure in terms of the direction taken by the negotiation process” (Druckman and Olekalns 2013b: 334). Whereas the departure is the reaction to the precipitant, the consequence is the direction the negotiation takes as a result of the departure (Druckman 2001, 2004). For instance, proposing a mutually beneficial offer after a period of no progress (departure) as a reaction to a social signal indicating that a redirection of the negotiation is required (precipitant) may direct the negotiation towards positive grounds or not (consequence). Thus, although departures are events that are potentially beneficial for the negotiation, their potential has to be realized by the negotiators.

As the characterization of precipitants, departures and consequences suggests, the three stages in Druckman’s (2001) turning point model are not realized unilaterally by a single negotiator. “Negotiators may choose to incorporate or ignore new ideas, they may accept or reject suggested procedural changes, and they may react or fail to react to external events” (Druckman et al. 2009: 15). In order to take into account the interactive nature of Druckman’s (2001) turning point model, we propose that the three stages are realized by interacts—interlocking action–reaction sequences in which negotiator A responds to the action of negotiator B and vice versa—that initiate and (dis)confirm the departure from zero-sum bargaining. Whether the departures have positive or negative consequences, the final step in Druckman’s (2001) model, depends on how negotiators enact the behavioral sequences.

We consider three interacts that introduce and conclude the turning point (see Fig. 1). The first interact, two steps before the turning point (TP_t−2_ to TP_t−1_), represents the precipitant in which negotiator B sends a social signal to negotiator A by reacting with an emotional change. The second interact (TP_t−1_ to TP_t_) constitutes the actual turning point in which negotiator A reacts to the social signal by proposing a more favorable offer compared to the previous offer by negotiator B. In the final interact (TP_t_ to TP_t+1_) negotiator B concludes the turning point by (dis)confirming the change introduced by A. According to our model, the (dis)confirmation occurs on two distinct levels. First, via social signals indicating (dis)approval of the progression on an emotional level and second by (mis)matching the turning point offer on a substantive level.

### Social Signals as Precipitants to Substantive Change

Turning points typically occur after a period of no progress, leaving negotiators prone to facing impasses, or after a period of crisis (Druckman 1986). This gives rise to precipitants that initiate the turning point. Following Druckman’s (2001) typology, precipitants can be either substantive or procedural, or have an internal or external source. External precipitants are events outside of the negotiation that cannot be directly controlled by the negotiators but nevertheless have an impact on the subsequent negotiation process (Druckman 2001; Druckman and Olekalns 2011). Internal precipitants, on the other hand, are deliberate actions undertaken by the involved parties themselves in order to change the progress of the negotiation. These actions may include procedural efforts addressing the structure or format of the negotiation such as putting a critical issue aside and postponing it to a later stage (Druckman and Olekalns 2011; Druckman et al. 2009). Substantive actions are precipitants that concern the issues at hand and consist of new ideas or concepts brought to the negotiation table by the involved parties (Druckman 2001), modifications and re-conceptualizations such as delinking issues (Druckman 2001; Druckman et al. 1991, 2009), reframing issues (Druckman and Olekalns 2012; Putnam and Fuller 2014), or introducing new multi-issue packages (Druckman 2001). These internal precipitants have garnered more interest in literature (Druckman et al. 2009) as they change the “flow” of the negotiation process (Donohue 2004).

Extending prior literature, we consider emotions as social signals that act as precipitants for substantive change (the departure). Accordingly, a social change at the emotional level is the precursor of substantive change at the offer level. Emotions are acute and intense psychophysiological changes in response to a significant event in a person’s environment (Rosenberg 1998). Compared to mood, emotions are of relatively short duration and are elicited by and directed to specific events, actions, or individuals (Frijda 1986; Lazarus 1991). Thus, they are also more likely to influence beliefs and behavior (Forgas 1992; Lazarus 1991). Research has established strong empirical evidence that emotions affect a negotiator’s own decisions and behavior, the reaction of the counterpart and, as a consequence, the outcome of the negotiation (for reviews, see, Druckman and Olekalns 2008; Griessmair et al. 2015; Olekalns and Druckman 2014).

Emotions permeate “virtually every aspect of organizational life, even those areas that have been traditionally thought of as the exclusive province of cognitive behavior” (Barsade and Gibson 2007: 51). They are “fundamental for understanding social behavior because they occur so frequently and play an important role in revealing how individuals regard themselves and respond to others” (Barry and Oliver 1996: 127). Negotiations are inherently emotional (Morris and Keltner 2000; Shapiro 2006) and “a natural setting for the study of affect” (Brief and Weiss 2002: 295) for two reasons. First, “(n)egotiation takes place within a relationship, a context in which emotions inevitably arise” (Shapiro 2002: 68). Second, negotiations always involve the frustration or achievement of goals; both are inherently related to emotional experience and expressions (Van Kleef et al. 2010). Thus, Shapiro (2006: 106) concludes that a ”negotiator cannot avoid emotions any more than he or she can avoid thoughts”. Similarly, Griessmair and Köszegi (2009) show that even if emotions are not expressed explicitly, the messages negotiators exchange always contain an emotional layer.

“(E)motional responses in a bargaining situation are hardly irrational disturbances” (Hegtvedt and Killian 1999: 270) but rather communicative acts that provide structure to social interaction by guiding, evoking, and motivating individuals’ actions (Keltner and Kring 1998; Morris and Keltner 2000). They shape the negotiation relationship and serve specific purposes during the interaction (Morris and Keltner 2000). In the context of Druckman’s (2001) model of turning points, we propose that emotions provide incentives and information to the counterpart (Morris and Keltner 2000; Van Kleef et al. 2010) that may serve as a precipitant to the departure as well as (dis)confirmation of the introduced change, thereby influencing whether negotiators move closer to or farther away from an agreement (the consequence).

Following the social-functional approach of emotions, negotiators’ emotional expressions have an important feedback, information, and signaling function (Barry et al. 2004; Griessmair 2017; Morris and Keltner 2000; Pietroni et al. 2008). They provide valuable information about negotiators’ willingness to agree, whether they approve of the counterpart’s behavior, how they perceive the status of the relationship, as well as about their attitudes and intentions (Daly 1991; Knutson 1996; Shapiro 2002). For instance, whereas the display of negative emotions may signal the importance of an issue or that a negotiator’s limits have been reached, showing positive emotions indicates that a negotiator is satisfied with the current development (Schroth et al. 2005; Van Kleef et al. 2010). Similarly, expressed emotions can also serve as positive or negative reinforcers of the counterpart’s behavior providing an incentive or deterrent to exhibit similar behavior in the future (Cacioppo and Gardner 1999; Fischer and Roseman 2007). For instance, displaying negative emotions serves as a disincentive that encourages the counterpart to alter his/her undesired behavior; positive emotions reward the counterpart for the shown behavior and increase the likelihood that s/he will exhibit similar behavior in the future.

Further, it is important to distinguish whether the information and incentive function of emotions addresses the negotiators’ goals or their relationship (Griessmair 2017; Kumar 1997; Markus and Kitayama 1991). Goal-oriented emotions such as pleasure and displeasure are related to the progress individuals make towards obtaining their objectives (Frijda 1986; Lazarus 1991). In the context of negotiations, this refers primarily to the economic and substantive goals (Griessmair 2017). Therefore, events that are considered detrimental for reaching a negotiator’s goal give rise to feelings of displeasure such as anger or frustration. In contrast, feelings of pleasure such as happiness or joy are likely to emerge when the events bring a negotiator closer to his/her goals (Van Kleef et al. 2010). Displaying these emotions signals to the counterpart whether her/his actions support or frustrate his/her negotiation partner’s goal and whether behavioral adjustment is required in order to reach an agreement (Barry et al. 2004; Morris and Keltner 2000). Other- versus self-oriented emotions, on the other hand, have the other individual rather than goal achievement as their primary referent and are associated with the success or failure in nurturing the relationship with the counterpart (Kumar 1997; Markus and Kitayama 1991). Thus, their information and incentive function addresses the perception of the relationship, whether a transgression in the relationship is perceived as such, the willingness to make amends, and the extent to which interdependence is disrupted or promoted (Griessmair 2017; Markus and Kitayama 1991; Van Kleef et al. 2010).

### Interacts (Dis)Confirming the Departure

Negotiators are highly dependent on each other in achieving their goals (Lewicki et al. 1999) and once a negotiator has performed actions to initiate a turning point, the counterpart may choose to either ignore the precipitant or react to it in a certain manner (Druckman et al. 2009). Thus, turning points are neither initiated nor concluded by a single negotiator. If and how negotiators react to the precipitant (cause) and depart from the previous behavioral pattern (turning point) will strongly influence in which outcome direction negotiations will move (consequence). Following the notion that social interactions are constituted not by individual behavior but by interlocking action–reaction sequences, negotiation researchers have treated interacts as the fundamental building blocks of negotiations (Brett et al. 2004). In the context of turning points, investigating actions in conjunction with their respective responses is especially informative: the type of behavioral sequences enacted by the parties reveal whether efforts to change the direction of the negotiations are made, whether these actions are (dis)confirmed by the counterpart, and whether stable patterns emerge or are disrupted (Brett et al. 1998, 2004; Olekalns et al. 2003).

Literature distinguishes between different forms of interacts in negotiation, each having specific consequences and functions in shaping the interaction (Brett et al. 2004; Donohue 1981; Olekalns and Smith 2000; Putnam and Jones 1982). In reciprocal sequences negotiators match each other’s moves. Responding in-kind provides an immediate reinforcement for a negotiator’s actions (Brett et al. 2004), signals a shared understanding of the negotiation (Putnam 1990) and, in consequence, stabilizes the ongoing interaction pattern. Although negotiators have a strong tendency to respond in kind (Butt et al. 2005), whether this matching behavior is beneficial or detrimental depends on the type of reciprocal sequences (Weingart and Olekalns 2004). Research has shown that reciprocating distributive tactics reinforces a distributive orientation and is more likely to lead to impasses; matching integrative tactics, on the other hand, confirms a shared integrative strategic perspective and promotes settlement and high joint gains (Brett et al. 1998; Olekalns and Smith 2000; Weingart et al. 1999, 1990). In nonreciprocal sequences negotiators mismatch each other’s behavior. This mismatching signals divergent perspectives and does not reinforce the preceding action (Brett et al. 2004), thus providing a stimulus for altering the dynamics of the negotiation (Brett et al. 1998; Olekalns and Smith 2000). In fact, negotiators reaching an agreement are more likely to use such transformational sequences (Olekalns and Smith 2000) in order to redirect the distributive orientation to positive grounds (Olekalns and Weingart 2003).

We apply the interact framework of action–reaction sequences to the emotional as well as substantive dimension of our model. Multi-criteria decision analysis provides the possibility to reflect the benefits of exchanged offers for each negotiator based on their preferences as represented by utility functions (Raiffa et al. 2002; Wallenius et al. 2008). Utility functions allow capturing the value of exchanged offers and counteroffers at the individual as well as at the dyad level. At the individual level, they represent the gain of the single negotiators. At the dyad level, the sum of both parties’ utilities reflect the joint gain achieved by the negotiators. When the joint gain increases over time, the negotiators have managed to create value during the process, increasing the pie rather than splitting it. The difference between the individual utilities constitutes the contract imbalance, a measure for the in(equity) of an offer or outcome widely used in negotiation research (Foroughi et al. 1995; Perkins et al. 1996). The higher the contract imbalance, the more an offer is disproportionately advantageous to one party (Delaney et al. 1997).

These measures are commonly used in negotiation research to describe the quality of exchanged offers and outcomes in negotiation research (Tripp and Sondak 1992) and can also be used to describe interaction steps between negotiators (Filzmoser and Vetschera 2008). We denote the utility of an offer made by party *A* to party *B* at time *t* by ${u}_{A,A}^{t}$ for party *A* (Own Utility) and by ${u}_{B,A}^{t}$ for party *B* receiving the offer (Other Utility). The joint utility of an offer proposed by party *A* to party *B* is given by ${{JU}_{B,A}^{t}={u}_{A,A}^{t}+u}_{B,A}^{t}$ and the contract imbalance by ${{CI}_{B,A}^{t}={u}_{A,A}^{t}-u}_{B,A}^{t}$. Negotiators engage in a transformational sequence when one party significantly changes the joint gain and/or the (in)equity compared to the counterpart’s previous offer, and in reciprocal sequences when the parties roughly match each other’s offers.

According to Druckman’s (2001) typology, departures can consist of procedural changes, incorporation of new ideas, or abandoning a give-and-take pattern (Druckman 2001). In the present study, we use abandoning a give-and-take pattern as the event constituting the turning point. The turning point marks the departure from zero-sum bargaining towards creating value and equal offers (Griessmair and Druckman 2018). This takes the form of a positive transformational interact: one negotiator significantly increases the joint utility ${JU}_{B,A}^{t}>{JU}_{A,B}^{t-1}$ and/or decreases the contract imbalance ${CI}_{B,A}^{t}<{CI}_{A,B}^{t-1}$ in response to the counterpart’s offer. Reciprocal sequences, on the other hand, consist of interacts in which the parties roughly match each other’s offers and counteroffers, that is ${JU}_{B,A}^{t}\approx {JU}_{A,B}^{t-1}$ and ${CI}_{B,A}^{t}\approx {CI}_{A,B}^{t-1}$. The sequences can be extended to double interacts, which consist of *A*’s behavior, *B*’s response, and *A*’s response to the behavior of *B* (Weick 1969). For instance, when a positive transformational sequence ${JU}_{B,A}^{t}>{JU}_{A,B}^{t-1}$ is followed by a reciprocal sequence ${JU}_{B,A}^{t}\approx {JU}_{A,B}^{t+1}$, negotiator *A*’s effort to change the direction of the negotiation is confirmed by negotiator *B*. Such a double interact reinforces the introduced departure (Brett et al. 2004), signals a shared understanding of the negotiation (Putnam 1990), and stabilizes the established positive direction towards a mutually beneficial agreement.

This general framework can also be applied to the social dimension. Whereas in transformational sequences negotiators react to the counterpart’s expressed emotions with a significant change in the level of their emotional expressions, reciprocal sequences consist of matching each other’s emotional display. For instance, engaging in a positive transformational sequence by responding with a significant increase of positive goal-oriented emotions compared to the other party’s goal-oriented emotions in the previous step reinforces the counterpart’s behavior (Cacioppo and Gardner 1999; Fischer and Roseman 2007) and signals that the negotiation is on the right track (Barry et al. 2004; Morris and Keltner 2000). Conversely, a negative transformational sequence—a significant decrease of positive goal-oriented emotions—serves as a negative reinforcer and feedback mechanism for counterproductive behavior that signals to the counterpart that a behavioral change is required (Cacioppo and Gardner 1999; Fischer and Roseman 2007; Morris and Keltner 2000).

**Fig. 2:**
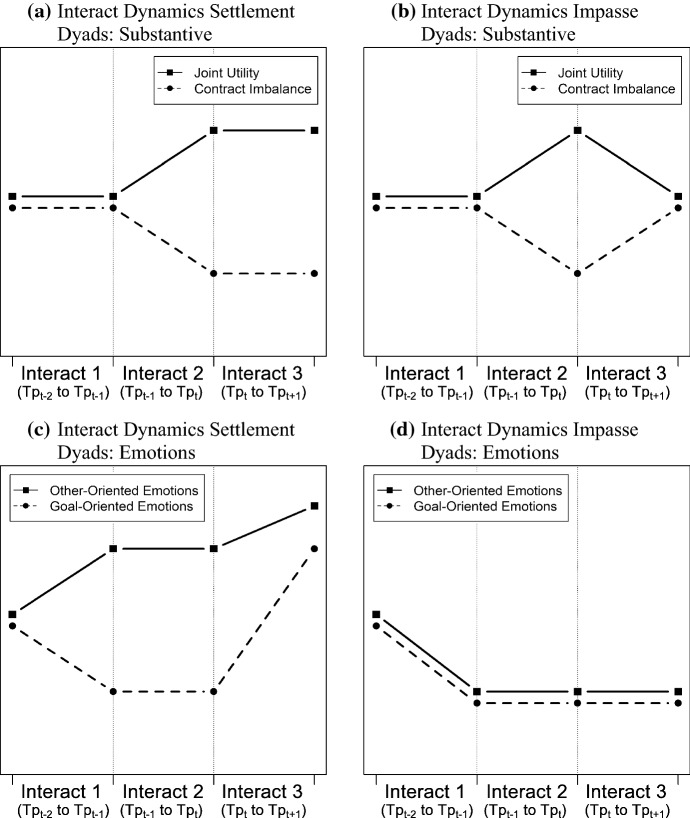
Proposed interact dynamics

The action–reaction sequences that precipitate and conclude the turning point for settlement as compared to impasse dyads are discussed in the subsequent section. Figure 2 provides an overview of the proposed interact dynamics.

## Hypotheses

### Precipitating the Turning Point

Turning points are typically preceded by a longer period in which negotiators make no progress towards a mutually beneficial agreement (Druckman 1986). A number of studies confirm that before starting to integrate and create value, negotiators engage in distributive value claiming—they assume that their goals are incompatible, make ambit claims, posture for position, and their argumentation focuses on their sources of power and perceived rights (Adair and Brett 2005; Lytle et al. 1999; Olekalns and Weingart 2003; Putnam 1990). When perpetuating this pattern of positional zero-sum bargaining, negotiators are likely to experience goal frustration and sense that an impasse is looming. Not moving closer to their desired objective, in turn, gives rise to negative emotions (Frijda 1986; Kumar 1997; Lazarus 1991). Prior research has shown that negotiators in a negative affect state have less mutual trust (Anderson and Thompson 2004), are less cooperative and have less concern for the counterpart (Forgas 1998; Yifeng et al. 2008).

We argue that the expression of negative emotions may also be beneficial as it serves as a precipitant to a turning point possibly steering the negotiation back to the right track. This view is supported by studies showing that the display of negative affect results in larger concessions by the counterpart (Van Kleef et al. 2004a, b). Also, when perceived as justified, negative emotions do not necessarily lead to retaliation (Van Kleef and Côté 2007) and especially in competitive settings they may induce the counterpart to be more cooperative (Griessmair 2017; Van Kleef et al. 2010). Based on their function as negative reinforcer and feedback mechanism for counterproductive behavior (Cacioppo and Gardner 1999; Fischer and Roseman 2007; Morris and Keltner 2000), we propose that the turning points are initiated by a negative transformational sequence of goal-oriented emotions that destabilizes the ongoing negotiation pattern and signals that a behavioral change is required (see first interact in Fig. 2c, d). Thereby, it provides a stimulus for altering the dynamics of the negotiation and acts as a precipitant to the turning point.

#### H1a

Turning points are precipitated by a negative transformational sequence of goal-oriented emotions.

The expression of negative emotions, however, does not only have informational and disincentive functions but can also have detrimental effects (Van Kleef and Côté 2007). Negotiators confronted with an opponent expressing negative affect are more likely to develop a negative impression of the counterpart (Van Kleef et al. 2004a, b) and are less likely to reach an agreement (Friedman et al. 2004; Kopelman et al. 2006) or to be willing to engage in future negotiations with the counterpart (Kopelman et al. 2006). Furthermore, negative emotions are likely to be reciprocated by the counterpart (Barsade 2002; Hatfield et al. 1994), particularly if they appear unjustified and both actors are equal in power (Van Kleef and Côté 2007). For instance, Friedman et al. (2004) show that the display of anger triggers an angry response by the counterpart, which, in turn, increases the likelihood of not reaching an agreement. This pattern of contentious reciprocation and its detrimental effects has been confirmed with regard to emotions in negotiations (Nielek et al. 2010) as well as with strategies and tactics (Brett et al. 1998; Olekalns and Smith 2000; Weingart et al. 1999, 1990).

Thus, introducing the departure via a negative transformational sequence of goal-oriented emotions may backfire. Prior research investigating when negative emotions have positive or negative effects has primarily focused on contextual variables such as justifiability, interdependence and power structure, and information processing needs (Van Kleef and Côté 2007). We propose that the potentially detrimental effects that may arise from introducing a departure via a negative transformational sequence of goal-oriented emotions cannot only be countered by contextual factors but also by coupling it with a positive transformational sequence of other-oriented emotions.

The suggested mechanism is akin to Fisher and Ury’s (1981) classic advice to separate the people from the problem and Brett et al.’s (1998) concept of mixed communication. Inspired by Fisher and Ury (1981), Steinel et al. (2008) show that negative behavior-oriented emotions—emotions directed towards “the problem” such as the counterpart’s offer—induced the counterpart to make concessions. Conversely, negative person-oriented emotions—emotions directed towards “the people”—had no such effect. Based on this study, Van Kleef and Côté (2007) conclude that while expressing negative emotions about a person’s offer seems to pay, expressing negative emotions at them personally may backfire.

While Steinel et al. (2008) have investigated person- and behavior-oriented emotions separately in an experimental setting, in actual negotiation interactions allowing for naturalistic communication negotiators can convey multiple ideas within a single speaking turn (Brett et al. 1998; Weingart and Olekalns 2004; Weingart et al. 2004). That is, they can pair negative goal-oriented emotions directed towards “the problem” with positive other-oriented emotions directed towards “the people”. Brett et al. (1998) refer to this strategy of conveying complementary messages that serve two purposes in a single speech act as mixed-communication. In their study investigating conflict cycles, they show that reciprocating with a contentious strategy while at the same time communicating cooperation is more effective than a uniform tit-for-tat strategy. The underlying rationale is that the contentious reciprocation without cooperative signals helps secure individual gain but at the same time is likely to result in conflict spirals and potentially lead to a breakdown of the negotiation (Brett et al. 1998; Olekalns and Smith 2000; Weingart et al. 1999, 1990). Conversely, reciprocating cooperatively without a contentious component is likely to signal weakness, leaving the negotiator prone to exploitation (Brett et al. 1998). Successful negotiators use both: they engage in contentious reciprocation in order not to be exploited, but at the same time communicate cooperation in order to prevent an escalation of the conflict (Brett et al. 1998). Following a similar rationale, Gibbons et al. (1992) advise negotiators to use ‘thromises’—a combination of threats and promises—rather than employing them individually. The effectiveness of mixed communication is also supported by controlled experimental evidence. Lindskold and Bennett (1973) conducted a prisoner’s dilemma study in which participants could choose to send a contentious message (a threat), a cooperative message (a promise), or both. Results show that combining the threat with the promise not only resulted in a more favorable evaluation of the sender but also in more cooperation.

We expect to observe a similar mechanism discriminating between successful and unsuccessful dyads with regard to social precipitants of turning points. A negotiator’s message contains several emotional layers simultaneously; even though they are not necessarily expressed directly and may vary in strength (Griessmair 2017; Griessmair and Koeszegi 2009). Controlled experiments investigating the role of emotions in negotiations employed direct emotional expressions such as “I am angry” or “I am happy” for their manipulations (e.g., Van Kleef et al. 2004a, b). Research investigating naturalistic negotiation interactions, however, found that emotions are also conveyed implicitly in natural negotiation communication. Conveying emotions does not necessarily require explicit emotional expressions, but negotiators associate distinct emotions with specific utterances and expressions (for a discussion and examples, see, Gibbons et al. 1992; Griessmair and Koeszegi 2009; Schroth et al. 2005).

For instance, consider the following interact before the departure (TP_t_). Negotiator A sends the message “Your prior proposal is not what we are looking for and we need to work on an agreement.” (TP_t−2_) to which negotiator B (TP_t−1_) reacts with one of the following two alternatives: (1) “Your current proposal is unacceptable and as we are a respected company providing high quality products we expect a better offer.” (2) “Your current proposal is unacceptable but I’m confident that if we work together on the problem we will be able to reach an agreement that is beneficial for both of us.”. Although not expressed directly, both messages (1) and (2) convey negative goal-oriented emotions (“your current proposal is unacceptable”) directed towards negotiator A’s offer (“the problem”). They serve as feedback mechanism for counterproductive behavior and signal that behavioral change at the substantive level is required (Cacioppo and Gardner 1999; Fischer and Roseman 2007; Morris and Keltner 2000). Message (1) combines this negative transformational sequence of goal-oriented emotions with a negative transformational sequence of other-oriented emotions (“as we are a respected company providing high quality products we expect a better offer.”). Thereby, negotiator B signals not only dissatisfaction at the substantive level but also conveys feelings of superiority and dominance, disengagement from the personal relationship, and a disruption of interdependence (Markus and Kitayama 1991; Russell and Mehrabian 1977). Conversely, message (2) conveys mixed emotional signals by combining the negative transformational sequence of goal-oriented emotions with a positive transformational sequence of other-oriented emotions (“I’m confident that if we work together on the problem we will be able to reach an agreement that is beneficial for both of us.”). Additionally to signal dissatisfaction on the substantive level, negotiator B simultaneously gives a positive outlook on the relational level by conveying feelings of connection, affiliativeness, and promoting interdependence (Markus and Kitayama 1991; Russell and Mehrabian 1977).

Drawing on Brett et al.’s (1998) model of conflict spirals, we propose that the successful dyads compensate the potential drawbacks of the negative transformational sequence of goal-oriented emotions by pairing it with a positive transformational sequence of other-oriented emotions. Whereas the former signals dissatisfaction with the current progress of the negotiation, the latter conveys affiliative intent and commitment for the relationship (Griessmair 2017; Kumar 1997; Markus and Kitayama 1991). Next to the substantive dimension, relationship building and establishing an appropriate working climate are cornerstones of successful negotiations (Moore et al. 1999; Poole et al. 1992). By using mixed transformational sequences as social signals, settlement dyads initiate change at the substantive level and at the same time maintain a favorable relationship. Conversely, we expect that dyads concluding the negotiation with a stalemate do not engage in regulatory behavior and use uniform social signals by pairing the negative transformational sequence of goal-oriented emotions with a negative transformational sequence of other-oriented emotions (see first interact in Fig. 2c, d). Rather than engaging in compensatory behavior by employing mixed social signals, they disrupt the process on a substantive as well as relational level, eventually increasing the likelihood of an impasse.

#### H1b

Successful negotiation dyads pair the negative transformational sequence of goal-oriented emotions with a positive transformational sequence of other-oriented emotions.

#### H1c

Unsuccessful negotiation dyads pair the negative transformational sequence of goal-oriented emotions with a negative transformational sequence of other-oriented emotions.

#### Concluding the Turning Point

According to our model, the social signal conveyed via transformational emotional sequences acts as a precipitant that leads to a departure from zero-sum bargaining after which the parties start making more economically favorable offers. Following Druckman’s (2001) framework, these turning points have positive or negative consequences depending on whether the changes are incorporated or not. Thus, once a party has introduced a favorable turning point, it has to be confirmed by the counterpart as a new stable pattern and dominant dynamic for the subsequent negotiation interaction (Druckman et al. 2009). In order to do so, we expect that in successful negotiations the counterpart matches the offer from the negotiator that has introduced the turning point (see third interact in Fig. 2a). This reciprocal sequence acts as an immediate reinforcement of the positive departure (Brett et al. 2004) and communicates shared understanding about the newly established direction (Putnam 1990).

##### H2a

In successful negotiations, the turning point is reinforced by a reciprocal sequence of joint utility and contract balance.

The progress the negotiators make towards a mutually beneficial agreement should also be reflected in their emotional experience and expressions. As the departure brings the negotiators closer to their goals, both on a substantial and on a relational level, positive emotions are likely to emerge (Frijda 1986; Lazarus 1991). Druckman et al. (2009) also show that parties with aligned goals are more likely to create a positive climate. Furthermore, concluding the departure with a positive transformational sequence of goal- and other-oriented emotions conveys satisfaction with the current development (Schroth et al. 2005; Van Kleef et al. 2004a, 2010) and rewards the desired behavior shown at the turning point (Cacioppo and Gardner 1999; Fischer and Roseman 2007). Thus, we propose that in successful negotiations the departure is not only confirmed on a substantive level by matching the turning point offer, but also with social signals (see third interact in Fig. 2c).

##### H2b

In successful negotiations, the turning point is concluded by a positive transformational sequence of goal- and other-oriented emotions.

Conversely, we expect that impasse dyads fail to reinforce the newly introduced dynamic on both an emotional and a substantive level. Previous research has found that successful negotiation dyads are more likely to use transformational sequences to steer the negotiation away from distributive bargaining (Olekalns and Smith 2000). However, transformational sequences do not only provide a stimulus for change (Brett et al. 1998; Olekalns and Smith 2000), but also signal divergent perspectives and fail to reinforce the preceding behavior (Brett et al. 2004). This kind of counterproductive mismatching occurs “when negotiators strategically utilize the information conveyed by the counterpart’s behavior to better serve their own interests” (Butt et al. 2005: 688). That is, they take advantage of a concessionary counterpart by hard value claiming and react to conciliatory behavior with high demands (Bateman 1980). By taking the counterpart’s coming closer behavior at the turning point as sign of weakness and countering it with mismatching, unsuccessful dyads disrupt the positive change resulting in negative consequences for the negotiation (cf., Druckman 2001) (see third interact in Fig. 2b).

##### H2c

In unsuccessful negotiations, the turning point is disconfirmed by a negative transformational sequence of joint utility and contract balance.

According to our model, the turning point is initiated by a negative transformational sequence consisting of a sharp emotional decline. Successful negotiators conclude the turning point with a positive transformational sequence of goal- and other-oriented emotions. Not only does this provide a reinforcement of the turning point behavior and signals goal-congruence (Cacioppo and Gardner 1999; Morris and Keltner 2000), but also helps to re-direct the negotiation towards positive emotional grounds after the decline that introduced the turning point. Stalemate dyads, on the other hand, are unlikely to experience progress towards an agreement that gives rise to positive emotions (Frijda 1986; Lazarus 1991). Thus, we expect that unsuccessful negotiators reinforce the emotional climate of the turning point with a reciprocal sequence of goal- and other-oriented emotions rather than introducing emotional change via a positive transformational sequence (see third interact in Fig. 2d).

##### H2d

In unsuccessful negotiations, the turning point is concluded by a reciprocal sequence of goal- and other-oriented emotions.

### Methods

#### Data and Sample Description

The data for the study were collected using the database from Inspire (Kersten and Noronha 1999). Inspire is a web-based electronic negotiation support system. Operational since 1995, it has been used extensively for teaching and research (e.g., Griessmair 2017; Hine et al. 2009; Kersten and Zhang 2003; Kersten and Lai 2007). Participants in Inspire negotiations conduct a dyadic multi-attribute, mixed-motive negotiation about the supply of bicycle parts, with one party being the buyer (Cypress Cycles) and the other party the seller (Itex Manufacturing). Participants receive a detailed role explanation; however, no directions regarding preferences, limits, or negotiation style are given. Thus, the setting allows for both integrative and distributive strategies and outcomes. In addition, both parties are informed that alternative buyers or sellers are available and they can terminate the negotiation without reaching an agreement.

We randomly selected 60 negotiation dyads from the database—30 settlement and 30 stalemate dyads. The 120 negotiators in our sample exchanged in total 487 offers with corresponding text messages. The sample size was based on prior studies investigating the effect of the negotiation process (Olekalns et al. 1996, 2005) and turning points (Druckman and Olekalns 2013a; Druckman et al. 2009) on negotiation outcomes. As will be described in more detail below, participants in Inspire negotiations perform a utility negotiation before starting the negotiations. The thus identified utility values are employed for the economic variables in our analyses—contract imbalance, individual and joint utility of an offer—as well as for determining the turning point. Furthermore, with each offer and counteroffer negotiators also exchanged text messages. We employ multidimensional scaling (MDS) to identify the emotional dimensions conveyed by these messages serving as social signals.

### Utility Elicitation and Definition of Turning Points

When using Inspire, participants are required to perform a utility evaluation of the issues and corresponding attributes. During this process, weights and values are assigned to the issues and corresponding attributes indicating their relative importance (for a more detailed description, see, Kersten and Noronha 1999). The thus elicited utilities allow evaluating the economic value of each offer exchange (joint utility, contract imbalance, and the individual utilities) as well as the negotiation outcome using Pareto efficiency.

The elicited utilities of both parties were also used to identify the turning point in a negotiation. On an economic level, turning points can be defined as abandoning a give-and-take pattern that marks the departure from zero-sum bargaining towards the exchange of more favorable offers for both parties (Druckman 2001; Griessmair and Druckman 2018). Thus, we used the strongest decline in contract imbalance over the entire negotiation as the turning point. A transformational sequence with a decrease in contract imbalance reflects a negotiator’s effort to increase the economic equity and coming closer to the counterpart. Six negotiations exhibited two or more declines of approximately equal magnitude over the entire negotiation. In these cases, the first decline was chosen as the turning point.

### Emotional Content of Offers

In order to measure the emotional content of the messages negotiators sent along with the offers, we employed multidimensional scaling (MDS).^2^ This procedure allows to capture the emotional layers expressed in messages and has already been used extensively in emotion (e.g., Feldman Barrett 2004; Russell 1980; White 2000) as well as negotiation research (e.g., Griessmair and Koeszegi 2009; Pinkley 1990; Pinkley et al. 1995). MDS is an attribute-free approach based on proximity judgements that uncovers the “hidden structure” of behavioral data and complex psychological phenomena by producing a spatial representation of the evaluated stimuli (Pinkley et al. 2005). Following the dimensional approach of emotions (Mauss and Robinson 2009), in MDS each message is located in a coordinate system of *n* independent emotional dimensions. That is, rather than assigning a single discrete emotion, each message is characterized by both other- and goal-oriented emotions with the loading of the message on the dimensions reflecting the strength of the conveyed emotion.

To generate the distance matrix serving as input for MDS we employed the subjective clustering method (Gelfand et al. 2001). It requires the independent raters to sort the messages into homogenous groups so that the messages in one group are emotionally similar to each other and dissimilar to the other groups. The frequency with which messages are sorted into the same group reflects their emotional proximity. The so obtained proximity matrix has been analyzed using PROXSCAL.

Following the inductive component of MDS, we used several criteria suggested in literature for determining the number of dimensions and their interpretation. This included verbal characterizations and ratings by the individuals performing the similarity judgments in the subjective clustering method, systematic comparisons of the messages loading high on the opposing pole of the dimensions, and regressing the dimensions on the negotiation outcome (Kruskal and Wish 1977; Perkins and Reynolds 1995; Pinkley et al. 2005). In two separate subsamples with different raters, the logistic regressions point to a 3-dimensional solution (Nagelkerke *R*^2^ = 0.37 and 0.40; Stress-I = 0.19 and 0.25). To assure that the findings are grounded in theory, the interpretation has been performed in light of existing emotional models (cf., Adair and Brett 2005).

#### Goal-Oriented Emotions (Pleasure–Displeasure)

The first dimension resembles pleasure–displeasure (Feldman Barrett 1998; Feldman 1995). This emotional dimension is related to the progress individuals make towards obtaining their objectives (Frijda 1986; Lazarus 1991). The messages that load high on the emotional positive pole have been characterized by the raters during the similarity judgements as ‘pleasant’, ‘content’, ‘happy’, and ‘excited’. Also the dominant theme of these messages reflects goal-congruency and the rate of progress made by the negotiators (e.g., “(…) we are extremely happy that you have responded very positively to our offer, the only difference of opinion between us, is in terms of price.”; “It seems we have reached a very good solution, now that I realize the importance of return policy to you.”) (Carver and Scheir 1990; Fischer and Van Kleef 2010; Kumar 1997; Van Kleef et al. 2010). Conversely, messages that load high on the negative pole of this dimensions have been described by the raters as ‘annoyed’, ‘frustrated’, ‘cold’, and ‘angry’. The messages express negative emotions as a result of not making progress towards the desired goal and holding the other party responsible for it (e.g., “You have gotten what you wanted on every issue. I can’t see where I have gained something.”; “I’m sorry but your offer is absolutely inacceptable. Please reconsider your offer.”) (Barclay et al. 2005).

#### Other- Versus Self-Oriented Emotion (Solidarity-Conflict and Submission-Dominance)

The MDS procedure resulted in two dimensions reflecting other- versus self-oriented emotions. Rather than being associated with goal achievement, both dimensions have the relationship with the counterpart as primary referent. They address success or failure in nurturing the relationship, the extent to which interdependence is disrupted or promoted, the willingness to make amends, and perceptions of the nature of the relationship (Griessmair 2017; Kumar 1997; Markus and Kitayama 1991).

The first other- versus self-oriented emotional dimension, solidarity-conflict or affiliativeness (Kitayama and Markus 1990; Markus and Kitayama 1991; White 2000), indicates the extent to which an individual is (dis)engaged from a personal relationship (Markus and Kitayama 1991). The messages loading high on the emotional positive pole of this dimension convey respect, feelings of connection, familiarity, and promote interdependence (Markus and Kitayama 1991) (e.g., “I am sure that our agreement is the best both of us could expect. It confirms my impression that our two companies are complementary and I have no doubt this opportunity is the beginning of a long collaboration”). Raters have characterized these messages as ‘understanding’, ‘optimistic’, ‘insightful’, or ‘compromising’. Conversely, messages on the opposing pole of this dimension have been denoted by the raters as ‘repellent’, ‘hostile’, ‘indifferent’, or ‘vengeful’ and express feelings that disrupt interdependence (e.g., “Our payment and return terms remain the same. My new offer is based in negotiations we are making with other potential suppliers.”).

The second dimension related to other- versus self-oriented emotions reflects the emotional dimension dominance versus submission or potency (Bush 1973; Neufeld 1975; Russell and Mehrabian 1977; White 2000). As opposed to solidarity-conflict, submission-dominance focuses on positioning the self in relation to the counterpart by conveying feelings that relate to the degree of superiority, control, and influence over the counterpart (Russell and Mehrabian 1977). Accordingly, messages loading high on the emotional negative pole of this dimension include a commanding tone, imposing conditions, and communicating one’s own expectations in a coercive manner (e.g., “We are providing you sophisticated and high precision metal parts (…) recognized corporation in this market for the last 50 years. (…) we’ll wait for an initial reasonable offer to start talking for real”). They have been characterized by the raters as ‘dominant’, ‘demanding’, ‘scornful’, and ‘disdainful’. Conversely, messages loading high on the positive pole have been described by the raters as ‘considerate’, ‘obliging’, and ‘agreeable’. They convey emotional repair work and signs of appeasement (Van Kleef et al. 2010) and express feelings of appreciation, willingness to take the other’s perspective, and making amends for a possible transgression (e.g., “We thought about your last offer and we will agree to the return condition because your objection concerning the high quality components is understandable-of course we are responsible for bad manufactured goods.”).

## Results

**Fig. 3:**
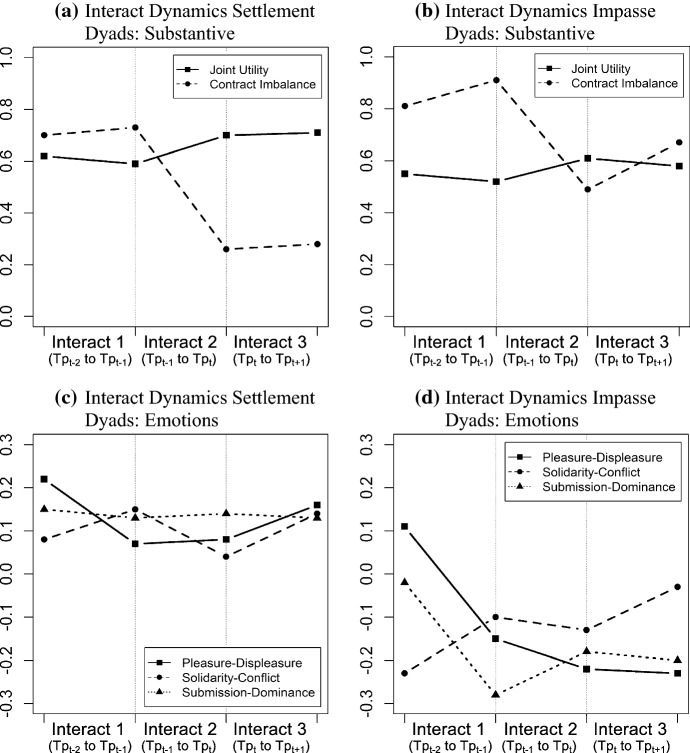
Interact dynamics

We submitted the elicited emotional dimensions and utility measures to 2 (agreement versus no agreement) × 2 (interact) ANOVAs, with repeated measures on the latter factor. A significant main effect for the interacts (within-subjects) indicates a transformational sequence, that is, the negotiators reacted to the counterpart with a significant change in behavior. When symmetry violations (Mauchly’s *W*) occurred we made the respective adjustments (Greenhaus–Geisser) to take into account inflated *F*-statistics (Bergh 1995). We further conducted planned post-hoc paired *t* tests in order to provide a more detailed picture of the interaction dynamics. Figure 3 displays the interaction dynamics for settlement and impasse dyads and Table 1 shows means and standard deviations for each interaction step. For comparability, the elicited emotional dimensions and utility measures were normed to 1.

**Table 1:** Mean (SD) of the interaction steps

		Interact (1)		Interact (2)	Interact (3)
		Actor A TP_t−2_	Actor B TP_t−1_	Actor A TP_t_	Actor B TP_t+1_
Own utility (OwU)	Impasse dyads	0.91 (0.13)	0.93 (0.09)	0.81 (0.12)	0.87 (0.10)
	Settlement dyads	0.93 (0.09)	0.91 (0.10)	0.78 (0.12)	0.81 (0.11)
Other utility (OtU)	Impasse dyads	0.13 (0.19)	0.04 (0.22)	0.34 (0.18)	0.20 (0.23)
	Settlement dyads	0.23 (0.23)	0.19 (0.23)	0.53 (0.20)	0.53 (0.16)
Joint utility (JU)	Impasse dyads	0.55 (0.07)	0.52 (0.13)	0.61 (0.08)	0.58 (0.12)
	Settlement dyads	0.62 (0.09)	0.59 (0.12)	0.70 (0.13)	0.71 (0.11)
Contract imbalance (CI)	Impasse dyads	0.81 (0.26)	0.91 (0.25)	0.49 (0.23)	0.67 (0.26)
	Settlement dyads	0.70 (0.29)	0.73 (0.27)	0.26 (0.21)	0.28 (0.18)
Pleasure–displeasure (PD)	Impasse dyads	0.11 (0.36)	− 0.15 (0.43)	− 0.22 (0.37)	− 0.23 (0.35)
	Settlement dyads	0.22 (0.30)	0.07 (0.29)	0.08 (0.21)	0.16 (0.23)
Solidarity-conflict (SC)	Impasse dyads	− 0.23 (0.38)	− 0.10 (0.39)	− 0.13 (0.38)	− 0.03 (0.44)
	Settlement dyads	0.08 (0.43)	0.15 (0.43)	0.04 (0.42)	0.14 (0.45)
Submission-dominance (SD)	Impasse dyads	− 0.02 (0.41)	− 0.28 (0.40)	− 0.18 (0.53)	− 0.20 (0.46)
	Settlement dyads	0.15 (0.49)	0.13 (0.40)	0.14 (0.39)	0.13 (0.44)

### Precipitating the Turning Point

#### Preliminary Analyses

Before testing the proposed hypotheses, we conducted preliminary analyses testing that the turning point (TP_t−1_ to TP_t_) indeed constitutes an interact with a significant departure from zero-sum bargaining and that before the turning point (TP_t−2_ to TP_t−1_) no such behavior can be observed.

Prior to the turning point (TP_t−2_ to TP_t−1_), the 2 × 2 ANOVA revealed a significant main effect for the interacts with regard to joint utility, *F*(1, 50) = 57.78, *p* < 0.001, *ηp*^2^ = 0.54, *P*_*o*_ = 0.96, contract imbalance, *F*(1, 50) = 55.56, *p* < 0.001, *ηp*^2^ = 0.53 *P*_*o*_ = 0.95, and other utility, *F*(1, 50) = 68.97, *p* < 0.001, *ηp*^2^ = 0.58, *P*_*o*_ = 0.98. Furthermore, the results also show significant group effects (successful vs. unsuccessful dyads) for joint utility, *F*(1, 50) = 16.09, *p* < 0.001, *ηp*^2^ = 0.39 *P*_*o*_ = 0.78, contract imbalance, *F*(1, 50) = 13.45, *p* < 0.001, *ηp*^2^ = 0.35 *P*_*o*_ = 0.67, and other utility, *F*(1, 50) = 15.77, *p* < 0.001, *ηp*^2^ = 0.39 *P*_*o*_ = 0.76, as well as significant interaction effects for joint utility, *F*(1, 50) = 24.53, *p* < 0.001, *ηp*^2^ = 0.49 *P*_*o*_ = 0.93, contract imbalance, *F*(1, 50) = 26.90, *p* < 0.001, *ηp*^2^ = 0.52, *P*_*o*_ = 0.95, and other utility, *F*(1, 50) = 30.66, *p* < 0.001, *ηp*^2^ = 0.55, *P*_*o*_ = 0.97. For own utility we found neither a significant interact, *F*(1, 50) = 0.02, *p* = 0.887, *ηp*^2^ = 0.00, *P*_*o*_ = 0.05, nor a main effect for group, *F*(1, 50) = 1.06, *p* = 0.355, *ηp*^2^ = 0.04, *P*_*o*_ = 0.06, and an interaction effect *F*(1, 50) = 0.95, *p* = 0.392, *ηp*^2^ = 0.04, *P*_*o*_ = 0.06. Post-hoc paired *t* tests revealed that successful dyads are characterized by reciprocal sequences (${JU}_{A,B}^{t-1}\approx {JU}_{B,A}^{t-2}$, *p* = 0.281, *d* = 0.29, *P*_*o*_ = 0.17; ${CI}_{A,B}^{t-1}\approx {CI}_{B,A}^{t-2}$, *p* = 0.675, *d* = 0.09, *P*_*o*_ = 0.07; ${OwU}_{A,B}^{t-1}\approx {OwU}_{B,A}^{t-2}$, *p* = 0.177, *d* = 0.16, *P*_*o*_ = 0.1; ${OtU}_{A,B}^{t-1}\approx {OtU}_{B,A}^{t-2}$, *p* = 0.608, *d* = 0.19, *P*_*o*_ = 0.12) and unsuccessful dyads exhibit negative transformational sequences for other utility (${OtU}_{A,B}^{t-1}<{OtU}_{B,A}^{t-2}$, *p* = 0.049, *d* = 0.40, *P*_*o*_ = 0. 26), a slight increase of contract imbalance approaching significance (${CI}_{A,B}^{t-1}>{CI}_{B,A}^{t-2}$, *p* = 0.055, *d* = 0.39, *P*_*o*_ = 0.25) and reciprocal sequences for joint utility (${JU}_{A,B}^{t-1}\approx {JU}_{B,A}^{t-2}$, *p* = 0.129, *d* = 0.32 *P*_*o*_ = 0.20) and own utility (${OwU}_{A,B}^{t-1}\approx {OwU}_{B,A}^{t-2}$, *p* = 0.524, *d* = 0.18, *P*_*o*_ = 0.12). Overall, the results show that the action–reaction sequence prior to the turning point indicates no attempt for inducing a positive redirection of the negotiation with impasse dyads even showing a tendency for increasing zero-sum bargaining (see first interact in Table 1 and Fig. 3a, b).

At the turning point (TP_t−1_ to TP_t_), however, both successful and unsuccessful dyads exhibit a positive transformational sequence for joint utility (settlement: ${JU}_{B,A}^{t}>{JU}_{A,B}^{t-1}$, *p* < 0.001, *d* = 0.92, *P*_*o*_ = 0.76; impasse: ${JU}_{B,A}^{t}>{JU}_{A,B}^{t-1}$, *p* = 0.002, *d* = 0.88, *P*_*o*_ = 0.73) and contract imbalance (settlement: ${CI}_{B,A}^{t}<{CI}_{A,B}^{t-1}$, *p* < 0.001, *d* = 1.99, *P*_*o*_ = 0.1; impasse: ${CI}_{B,A}^{t}<{CI}_{A,B}^{t-1}$, *p* < 0.001, *d* = 1.77, *P*_*o*_ = 0.1) as well as a significant increase of other utility (settlement: ${OtU}_{B,A}^{t}>{OtU}_{A,B}^{t-1}$, *p* < 0.001, *d* = 1.64, *P*_*o*_ = 0.99; impasse: ${OtU}_{B,A}^{t}>{OtU}_{A,B}^{t-1}$, *p* < 0.001, *d* = 1.48, *P*_*o*_ = 0.98) and a significant decrease of own utility (settlement: ${OwU}_{B,A}^{t}<{OwU}_{A,B}^{t-1}$, *p* < 0.001, *d* = 1.26, *P*_*o*_ = 0.94; impasse: ${OwU}_{B,A}^{t}<{OwU}_{A,B}^{t-1}$, *p* < 0.001, *d* = 1.16, *P*_*o*_ = 0.91). Thus, we find a clear departure from zero-sum bargaining at the turning point after an action–reaction sequence of economically unfavorable offers. These results are confirmed by the 2 × 2 ANOVA that revealed significant interacts for joint utility, *F*(1, 56) = 72.91, *p* < 0.001, *ηp*^2^ = 0.57, *P*_*o*_ = 0.97, contract imbalance, *F*(1, 56) = 173.63, *p* < 0.001, *ηp*^2^ = 0.76, *P*_*o*_ = 1.0, own utility, *F*(1, 56) = 15.13, *p* < 0.001, *ηp*^2^ = 0.21, *P*_*o*_ = 0.35, and other utility, *F*(1, 56) = 141.51, *p* < 0.001, *ηp*^2^ = 0.72, *P*_*o*_ = 1.0. Except for own utility, however, the ANOVA also reveals significant main effects for group [*JU*: *F*(1, 56) = 18.24, *p* < 0.001, *ηp*^2^ = 0.39, *P*_*o*_ = 0.83; *CI*: *F*(1, 56) = 21.94, *p* < 0.001, *ηp*^2^ = 0.44, *P*_*o*_ = 0.90; *OtU*: *F*(1, 56) = 23.65, *p* < 0.001, *ηp*^2^ = 0.46, *P*_*o*_ = 0.95] and significant interaction effects [*JU*: *F*(1, 56) = 17.70, *p* < 0.001, *ηp*^2^ = 0.39, *P*_*o*_ = 0.93; *CI*: *F*(1, 56) = 19.66, *p* < 0.001, *ηp*^2^ = 0.41, *P*_*o*_ = 0.95; *OtU*: *F*(1, 56) = 22.78, *p* < 0.001, *ηp*^2^ = 0.45, *P*_*o*_ = 0.97]. Thus, while both successful and unsuccessful dyads move towards value creation, successful dyads make a more favorable departure from zero-sum bargaining (see second interact in Table 1 and Fig. 3a, b).

#### *Emotional Precipitants (TP*_*t−2*_* to TP*_*t−1*_*)*

We predicted that this departure from zero-sum bargaining is initiated by a negative transformational sequence of goal-oriented emotions that destabilizes the ongoing negotiation pattern via its signaling and negative incentive function (H1a). The ANOVA reveals an interact approaching significance, *F*(1, 50) = 3.48, *p* = 0.068, *ηp*^2^ = 0.06, *P*_*o*_ = 0.07, and the post-hoc paired *t* tests show that both successful (${PD}_{A,B}^{t-1}<{PD}_{B,A}^{t-2}$, *p* = 0.023, *d* = 0.51, *P*_*o*_ = 0.33) and unsuccessful (${PD}_{A,B}^{t-1}<{PD}_{B,A}^{t-2}$, *p* = 0.009, *d* = 0.68, *P*_*o*_ = 0.52) dyads show a significant decline of goal-oriented emotions. The results resemble the pattern proposed in Fig. 2c, d (first interact)—an interact consisting of a negative emotional reaction to the counterpart right before the turning point. The ANOVA further shows no significant interaction effect, *F*(1, 50) = 0.69, *p* > 0.506, *ηp*^2^ = 0.02, *P*_*o*_ = 0.05, however, a significant main effect for group, *F*(1, 50) = 5.11, *p* < 0.001, *ηp*^2^ = 0.16, *P*_*o*_ = 0.19. While both impasse and settlement dyads decrease goal-oriented emotions, the former exhibit a more negative climate at the turning point (see first interact in Table 1 and Fig. 3c, d).

We further have hypothesized that dyads reaching an agreement would employ mixed communication and pair the negative transformational sequence of goal-oriented emotions with a positive transformational sequence of other-oriented emotions in order to signal commitment to the relationship and affiliative intent (H1b). The ANOVA revealed no significant interact for solidarity-conflict *F*(1, 50) = 0.43, *p* = 0.515, *ηp*^2^ = 0.01, *P*_*o*_ = 0.05, and submission-dominance *F*(1, 50) = 0.04, *p* = 0.834, *P*_*o*_ = 0.05. Contrary to our expectations, successful dyads do not engage in a transformational sequence, however, they still maintain the level of other-oriented emotions via reciprocal sequences (${SC}_{A,B}^{t-1}\approx {SC}_{B,A}^{t-2}$, *p* = 0.731, *d* = 0.16, *P*_*o*_ = 0.10; ${SD}_{A,B}^{t-1}\approx {SD}_{B,A}^{t-2}$, *p* = 0.865, *d* = 0.04, *P*_*o*_ = 0.06). Thus, no negative emotional signals on the relational level are conveyed. Impasse dyads, on the other hand, not only combine the negative transformational sequence of goal-oriented emotions with a matching sequence of solidarity-conflict (${SC}_{A,B}^{t-1}\approx {SC}_{B,A}^{t-2}$, *p* = 0.308, *d* = 0.34, *P*_*o*_ = 0.21) but also with a negative transformational sequence of submission-dominance (${SD}_{A,B}^{t-1}<{SD}_{B,A}^{t-2}$, *p* = 0.026, *d* = 0.64, *P*_*o*_ = 0.48). Thus, by reacting with a decline in other-oriented emotions they pair the emotional signal addressing goal achievement with a negative emotional signal addressing the status of the relationship (*H1c*). The ANOVA also revealed significant group effects for other-oriented emotions [*SC*: *F*(1, 50) = 6.80, *p* < 0.001, *ηp*^2^ = 0.20, *P*_*o*_ = 0.28; *SD*: *F*(1, 50) = 6.76, *p* < 0.002; *ηp*^2^ = 0.20, *P*_*o*_ = 0.28]. Similar to goal-oriented emotions, impasse dyads exhibit a more negative emotional climate at the turning point—also with regard to other-oriented emotions (see first interact in Table 1 and Fig. 3c, d).

In order to corroborate our results, we also performed regression analyses assessing the effect of the decline in goal-oriented emotions on contract imbalance and joint utility at the turning point and calculated the conditional effects of the likelihood goal-oriented emotions at the turning point have on reaching an agreement contingent on the level of other oriented-emotions.

For the regression analyses, we calculated the difference of the emotion variables (TP_t−2_ to TP_t−1_) and the utility variables (TP_t−1_ to TP_t_). The difference of the emotion variables reflects the change of emotional tone prior to the turning point with a higher positive value indicating a stronger decline in goal- and other-oriented emotions. According to our theorizing, the decline in goal-oriented emotions should be associated with an increase in joint utility (positive coefficient in the regression analyses) and a decrease in contract imbalance (negative coefficient in the regression analyses). Results of the regression analysis show that the decline of goal-oriented emotions is significantly associated with the decrease in contract imbalance (*β* = − 0.147, *p* = 0.033) and, with a *p*-value of *p* = 0.055, the increase in joint utility (*β* = 16.987) at the turning point, also when controlling for the joint utility (CI_Δ_: *β* = 0.000, *p* = 0.984; JI_Δ_: *β* = 0.343, *p* = 0.282) and the contract imbalance (CI_Δ_: *β* = − 0.227, *p* = 0. 149; JI_Δ_: *β* = 32.273, *p* = 0.112) of the offers exchanged prior to the departure. Thus, the results indicate that decreasing goal-oriented emotions before the turning point is associated with mutually coming closer as expressed by the contract imbalance. The other-oriented emotions are significantly associated neither with the increase in joint utility (SC: *β* = − 6.071, *p* = 0.346; SD: *β* = − 9.617, *p* = 0.149) nor the decrease in contract imbalance (SC: *β* = 0.033, *p* = 0.510; SD: *β* = 0.009, *p* = 0.855) at the turning point. Note that other-oriented emotions, having the relationship as primary referent, are not expected to affect the substantive value of offers. However, they should be related to whether negotiators reach an agreement or not, compensating on a relational level for the potential detrimental effects of decreasing goal-oriented emotions.

In order to corroborate the compensating effect of other-oriented emotions, we calculated the conditional effects of the likelihood that goal-oriented emotions at the turning point have on reaching an agreement contingent on other-oriented emotions. Results of the logistic regression show that a higher joint utility (*β* = 0.041, *p* = 0.036) and a lower contract imbalance (*β* = − 4.559, *p* = 0.008)^3^ of the turning point offer increases the likelihood that the parties reach an agreement. Furthermore, both pleasure–displeasure (*β* = 3.638, *p* = 0.010) and dominance-submission (*β* = 1.635, *p* = 0.033)^4^ are positively related to reaching an agreement. Moreover, an inspection of the conditional effects (see Table 2) reveals that the effect of the goal-oriented emotion pleasure on reaching an agreement becomes stronger with increasing other-oriented emotions, suggesting that the effect of goal-oriented emotions becomes stronger when simultaneously conveying higher levels of other-oriented emotions.

**Table 2:** Conditional effect of PD on agreement contingent on the value of SD

Percentile and value of SD		Conditional effect of PD	*p*
10th	− 0.779	2.137 (2.039)	0.295
25th	− 0.371	3,066 (1.366)	0.025
50th	0.033	3.986 (1.480)	0.007
75th	0.368	4.749 (2.102)	0.024
90th	0.572	5.212 (2.576)	0.043

Overall, we find support for the interaction dynamics depicted in Fig. 2. For both settlement and impasse dyads, the turning point is preceded by a negative transformational sequence of goal-oriented emotions (H1a). This is also supported by the regression analyses showing that the decline of goal-oriented emotions is associated with a decrease in contract imbalance. Impasse dyads combine the negative transformational sequence of goal-oriented emotions with a negative transformational sequence of other-oriented emotions (H1c). Contrary to our expectations, settlement dyads did not increase other-oriented emotions (H1b). However, rather than decreasing other-oriented emotions simultaneously with goal-oriented emotions, as can be observed in impasse dyads, they hold them constant and pair the decline in goal-oriented emotions with positive other-oriented emotions. In conjunction with the results of the logistic regression and the conditional effects, the findings indicate that the compensatory role of other-oriented emotions is conducive for reaching an agreement.

### Concluding the Turning Point

Following Druckman’s (2001) model, we hypothesized that the action–reaction sequences connected to the turning point (TP_t_ to TP_t+1_) (dis)confirm the departure and ultimately direct the negotiation towards or away from reaching a mutually satisfactory agreement (the consequence). As shown in Fig. 2 (third interact), we expected that as opposed to settlement dyads, unsuccessful dyads fail to reinforce the introduced change via reciprocal and positive transformational sequences.

#### *Economic (Dis)Confirmation of the Turning Point (TP*_*t*_* to TP*_*t+1*_*)*

For the economic dimensions, the ANOVA revealed significant interacts for joint utility, *F*(1, 57) = 16.51, *p* < 0.001, *ηp*^2^ = 0.22, *P*_*o*_ = 0.38, contract imbalance, *F*(1, 57) = 11.86, *p* < 0.001, *ηp*^2^ = 0.17, *P*_*o*_ = 0.24, and other utility, *F*(1, 57) = 17.57, *p* < 0.001, *ηp*^2^ = 0.24, *P*_*o*_ = 0.41. Furthermore, the results show significant group effects (successful vs. unsuccessful dyads) for joint utility, *F*(1, 57) = 17.26, *p* < 0.001, *ηp*^2^ = 0.38, *P*_*o*_ = 0.79, contract imbalance, *F*(1, 57) = 29.99, *p* < 0.001, *ηp*^2^ = 0.51, *P*_*o*_ = 0.96, and other utility, *F*(1, 57) = 29.86, *p* < 0.001, *ηp*^2^ = 0.51, *P*_*o*_ = 0.96, as well as significant interaction effects for joint utility, *F*(1, 57) = 8.79, *p* < 0.001, *ηp*^2^ = 0.24, *P*_*o*_ = 0.41, contract imbalance, *F*(1, 57) = 4.81, *p* = 0.012, *ηp*^2^ = 0.14, *P*_*o*_ = 0.18, and other utility, *F*(1, 57) = 7.65, *p* < 0.001, *ηp*^2^ = 0.21, *P*_*o*_ = 0.34. For own utility no significant interact, *F*(1, 57) = 0.03, *p* = 0.854, *ηp*^2^ = 0.00, *P*_*o*_ = 0.05, group effect, *F*(1, 57) = 2.20, *p* = 0.120, *ηp*^2^ = 0.07, *P*_*o*_ = 0.07, and interaction effect, *F*(1, 57) = 1.26, *p* = 0.291, *ηp*^2^ = 0.04, *P*_*o*_ = 0.06, was found. Overall, the ANOVA shows that impasse and settlement dyads exhibit different interaction patterns to conclude the turning point. The paired *t* tests further support these results and provide a more detailed picture. As predicted (H2a), in successful negotiations the turning point is concluded by reciprocal sequences of joint utility (${JU}_{A,B}^{t+1}\approx {JU}_{B,A}^{t}$, *p* = 0.472, *d* = 0.12, *P*_*o*_ = 0.09) and contract imbalance (${CI}_{A,B}^{t+1}\approx {CI}_{B,A}^{t}$, *p* = 0.733, *d* = 0.09, *P*_*o*_ = 0.08) as well as own utility (${OwU}_{A,B}^{t+1}\approx {OwU}_{B,A}^{t}$, *p* = 0.354, *d* = 0.22, *P*_*o*_ = 0.51) and other utility (${OtU}_{A,B}^{t+1}\approx {OtU}_{B,A}^{t}$, *p* = 0.987, *d* = 0.00, *P*_*o*_ = 0.05) (see Fig. 3a, third interact). Thereby, the negotiators signal a shared understanding of the introduced change (Putnam 1990) and reinforce the departure towards creating value initiated at the turning point (Brett et al. 2004).

Conversely, impasse dyads fail to reinforce the positive departure as predicted (H2c) and conclude the turning point by increasing the contract imbalance with a negative transformational sequences (${CI}_{A,B}^{t+1}>{CI}_{B,A}^{t}$, *p* = 0.001, *d* = 0.73, *P*_*o*_ = 0.59) (see Fig. 3b, third interact). Although, contrary to our prediction, they employ a reciprocal sequence with regard to joint utility (${JU}_{A,B}^{t+1}\approx {JU}_{B,A}^{t}$, *p* = 0.932, *d* = 0.02, *P*_*o*_ = 0.25), inspecting the individual utilities tells a different story. Whereas successful dyads conclude the turning point with matching the individual utilities, impasse dyads counter the turning point offer by increasing the own utility (${OwU}_{A,B}^{t+1}>{OwU}_{B,A}^{t}$, *p* = 0.011, *d* = 0.58, *P*_*o*_ = 0.43) and decreasing the other utility (${OtU}_{A,B}^{t+1}<{OtU}_{B,A}^{t}$, *p* = 0.005, *d* = 0.660, *P*_*o*_ = 0.52). By employing negative transformational sequences and countering with zero-sum bargaining, they disrupt the redirection towards creating value introduced at the turning point and disconfirm the change.

#### *Emotional (Dis)Confirmation of the Turning Point (TP*_*t*_* to TP*_*t+1*_*)*

The emotional dimensions show a similar picture (see third interact in Table 1 and Fig. 3c, d). The ANOVA revealed significant interacts for pleasure–displeasure, *F*(1, 57) = 4.17, *p* = 0.046, *ηp*^2^ = 0.07, *P*_*o*_ = 0.08, and submission-dominance, *F*(1, 57) = 5.55, *p* < 0.022, *ηp*^2^ = 0.09, *P*_*o*_ = 0.1, as well as significant group effects for pleasure–displeasure, *F*(1, 57) = 21.82, *p* < 0.001, *ηp*^2^ = 0.43, *P*_*o*_ = 0.89, and submission-dominance, *F*(1, 57) = 6.65, *p* = 0.003, *ηp*^2^ = 0.19, *P*_*o*_ = 0.28. The interaction effects are not significant for pleasure–displeasure, *F*(1, 57) = 1.92, *p* = 0.156, *ηp*^2^ = 0.06, *P*_*o*_ = 0.07, and approach significance for submission-dominance, *F*(1, 57) = 2.81, *p* < 0.068, *ηp*^2^ = 0.09, *P*_*o*_ = 0.1. For solidarity-conflict no significant interact, *F*(1, 57) = 0.13, *p* = 0.718, *ηp*^2^ = 0.00, *P*_*o*_ = 0.05, group effect, *F*(1, 57) = 2.03, *p* = 0.141, *ηp*^2^ = 0.07, *P*_*o*_ = 0.08, and interaction effect, *F*(1, 57) = 0.62, *p* = 0.541, *ηp*^2^ = 0.02, *P*_*o*_ = 0.05, was found. Paired *t* tests reveal that, consistent with our hypothesis (*H2d*), unsuccessful dyads match the emotional climate that precipitated the turning point with reciprocal sequences (${PD}_{A,B}^{t+1}\approx {PD}_{B,A}^{t}$, *p* = 0.932, *d* = 0.02, *P*_*o*_ = 0.06; ${SC}_{A,B}^{t+1}\approx {SC}_{B,A}^{t}$, *p* = 0.296, *d* = 0.25, *P*_*o*_ = 0.16; ${SD}_{A,B}^{t+1}\approx {SD}_{B,A}^{t}$, *p* = 0.842, *d* = 0.05, *P*_*o*_ = 0.07) rather than introducing emotional change with positive transformational sequences. Contrary to our prediction (H2b), we also find reciprocal rather than positive transformational sequences for successful dyads (${PD}_{A,B}^{t+1}\approx {PD}_{B,A}^{t}$, *p* = 0.249, *d* = 0.35, *P*_*o*_ = 0.24; ${SC}_{A,B}^{t+1}\approx {SC}_{B,A}^{t}$, *p* = 0.385, *d* = 0.22, *P*_*o*_ = 0.15; ${SD}_{A,B}^{t+1}\approx {SD}_{B,A}^{t}$, *p* = 0.960, *d* = 0.01, *P*_*o*_ = 0.05). However, successful dyads exhibit a more positive emotional climate during and after the turning point as evident by the significant group effects. They did not exhibit the decline in other-oriented emotions as observable in impasse dyads and additionally, when considering one more interact, an upwards trend of goal-oriented emotions is clearly observable (${PD}_{A,B}^{t+2}>{PD}_{B,A}^{t+1}$, *p* = 0.063, *d* = 0.48, *P*_*o*_ = 0.34; ${PD}_{B,A}^{t+2}>{PD}_{B,A}^{t}$, *p* = 0.021, *d* = 0.84, *P*_*o*_ = 0.1).

Overall, the results support the proposed interaction dynamics (see Fig. 2). Whereas successful dyads employ reciprocal sequences confirming the departure from zero-sum bargaining introduced at the turning point, impasse dyads exhibit negative transformational sequences disrupting the redirection towards creating value (H2a and H2c). While we expected positive transformational sequences from successful dyads (H2b) and reciprocal sequences from unsuccessful dyads (H2d), both use matching sequences to conclude the turning point. Yet, settlement dyads reciprocate at a more positive emotional level and an upwards trend for goal-oriented emotions is observable.

## Discussion

Turning points may be decisive in determining whether the parties involved in a dispute reach a mutually satisfactory solution or end up in an impasse (Donohue 2004; Druckman 2001; Druckman and Olekalns 2011). The aim of the present study was to examine theoretically and empirically the question of how breakpoint profiles differ between successful and unsuccessful dyads. Based on Druckman’s (2001) model, we introduced social signals as a new class of precipitants and integrated action–reaction sequences that initiate and (dis)confirm the departure from zero sum bargaining. Our results show that whether the turning point results in a positive consequence, a final settlement, is affected by how negotiators enact these behavioral sequences. Overall, the present article contributes to our understanding of how negotiators can successfully initiate and conclude departures and provides novel insights into the underlying emotional mechanisms.

Previous research on turning points has shown that negotiators can initiate departures potentially directing the negotiation towards positive grounds by taking procedural and substantive actions (Druckman 2001; Druckman et al. 1991, 2009; Putnam and Fuller 2014). Our findings show that social signals can serve a similar function by acting as precursors to economic change at the offer level and introduce a departure from zero-sum bargaining—in both successful and unsuccessful dyads the departure from zero-sum bargaining towards creating value is preceded by a negative transformational sequence of goal-oriented emotions. In line with prior research (e.g., Morris and Keltner 2000; Van Kleef et al. 2004a, b), the results confirm that negotiators can introduce a stimulus for change, destabilize the ongoing negotiation pattern, and signal that behavioral change is required by reacting with a decline in goal-oriented emotions.

However, in order to introduce the required change without endangering the outcome of the negotiation, it seems crucial that negotiators compensate the decline in goal-oriented emotions on a relational level rather than pairing the negative transformational sequence of goal-oriented emotions with a negative transformational sequence of other-oriented emotions. Prior research has shown that expressing negative emotions in negotiations may have serious detrimental effects (e.g., Friedman et al. 2004; Kopelman et al. 2006; Van Kleef et al. 2004a; b) and studies investigating when expressing negative emotions helps or hurts in negotiations have primarily focused on contextual factors (for a review, see, Van Kleef and Côté 2007). Based on Brett et al.’s (1998) concept of mixed-communication, our findings show that negotiators can initiate the departure and simultaneously reduce the risk of jeopardizing a final agreement by conveying complementary social signals that serve two purposes in a single speech act. Akin to Fisher and Ury’s (1981) classic advice to separate the people from the problem, settlement dyads pair the negative transformational sequence of goal-oriented emotions precipitating the departure with reciprocal sequences of other-oriented emotions. Using mixed social signals, settlement dyads compensate for the potential drawbacks of the negative transformational sequence by simultaneously conveying affiliative intent. Thereby, they signal dissatisfaction with the current progress of the negotiation and initiate change at the substantive level but at the same time signal commitment for maintaining a favorable relationship. Conversely, impasse dyads send a uniform social signal by pairing the negative transformational sequence of goal-oriented emotions with a negative transformational sequence of dominance. In doing so, they do not only lack the regulatory mechanism of mixed signals cushioning the potential conflict spiral (Brett et al. 1998) but also signal disengagement from the personal relationship and disruption of interdependence (Markus and Kitayama 1991), potentially harming the subsequent interaction (Moore et al. 1999; Poole et al. 1992).

Following Druckman’s (2001) model, the aforementioned precipitants initiate the turning point. Yet, whether this departure results in a settlement, the consequence, depends on whether the involved parties (dis)confirm the introduced change. Previous theorizing on turning points has highlighted that departures are not realized unilaterally (Druckman et al. 2009). Furthermore, Druckman and Olekalns (2013b) identify synchronization as one of the key mechanisms that trigger turning points. To the best of our knowledge, this is the first study investigating (dis)confirmatory action–reaction behavior as a crucial factor in turning point behavior that discriminates between impasse and settlement dyads. Our results show that both successful and unsuccessful dyads exhibit a turning point in which one party introduces a departure from zero-sum bargaining by decreasing contract imbalance and increasing joint gain. However, impasse and settlement dyads differ with regard to the action–reaction sequences following the departure, both on a substantive and an emotional level.

In settlement dyads, negotiators match their counterpart’s turning point offer in terms of contract imbalance, joint utility, and own- and other-utility. By employing reciprocal sequences, the negotiators reinforce the departure, signal shared understanding, and confirm the newly established pattern (Brett et al. 1998; Olekalns and Smith 2000; Putnam 1990; Weingart et al. 1999, 1990). In doing so, they direct the negotiation towards settlement (Druckman and Olekalns 2013b). The confirmation occurs not only on a substantive but also on an emotional level. By employing reciprocal sequences of other-oriented emotions, negotiators reaching an agreement convey affiliative intent and confirm the relational pattern established at the turning point. Furthermore, when considering one more interact, they show a clear upward trend in goal-oriented emotions signaling satisfaction with the development of the negotiation (Schroth et al. 2005; Van Kleef et al. 2010).

Conversely, negotiators that failed to reach an agreement counter the turning point offer by increasing their own utility and decreasing the other’s utility, resulting in a rise of the contract imbalance. Thus, rather than reinforcing the newly established mutually beneficial pattern, the negative transformational sequences disrupt the departure and disconfirm the change introduced at the turning point (Brett et al. 1998; Druckman and Olekalns 2013b; Olekalns and Smith 2000; Putnam 1990; Weingart et al. 1999, 1990). Furthermore, negotiators that ended the negotiation in an impasse also fail to redirect the negotiation back to positive emotional grounds after the departure—impasse dyads exhibit a decline of other- and goal-oriented emotions when introducing the turning point via negative transformational sequences and extend this pattern with reciprocal sequences after the departure.

Overall, the following key findings and advice for negotiators emanate from the systematic comparison of interaction steps initiating and concluding the turning point between settlement and impasse dyads. Although negative emotions are often considered detrimental in negotiations, expressing negative goal-oriented emotions may aid in initiating the departure as they clearly signal dissatisfaction with the current progress and that change is required. However, it appears to be crucial to pair the negative transformational sequence of goal-oriented emotions precipitating the departure with positive other-oriented emotions in order to signal affiliative intent and not disrupt the relationship between the negotiators. Once a negotiator has introduced the departure from zero-sum bargaining towards creating value and equal offers, it is key that the counterpart confirms this newly established pattern by reciprocating with an equally favorable offer for both parties. Not responding in kind to the turning point offer via a reciprocal sequence may jeopardize a successful, mutually satisfactory conclusion of the negotiation.

### Limitations and Outlook

In line with related studies and theorizing (Brett et al. 1998; Fisher and Ury 1981; Gelfand et al. 2006; Gibbons et al. 1992), including experimental studies allowing for causal inference (Lindskold and Bennett 1973; Steinel et al. 2008), our findings indicate that mixed emotional expressions in initiating the departure discriminates between successful and unsuccessful dyads. However, this article and the related research leaves open the question of why and when negotiators use mixed communication. A potential explanation may be relational concerns (Gelfand et al. 2006) or social motives (Weingart et al. 2007) that negotiators have prior to entering the negotiation. Negotiators that have low relational concern and pursue individualistic social motives may focus on “the problem” and express negative goal-oriented emotions without taking into account that other-oriented emotions directed at the relationship are crucial as well. Similarly, the capability of perceiving, understanding, and employing emotions—commonly summarized as emotional intelligence (Joseph and Newman 2010)—may critically affect how negotiators use and interpret the social signals initiating the departure. Investigating how relational concerns, social motives, and emotional intelligence affect the enactment of turning points may be an interesting avenue for future research.

Furthermore, prior research has shown that the epistemic motivation and information processing needs (Van Kleef et al. 2004b) as well as the interdependence structure in terms of the relative power of the parties and their alternatives (Sinaceur and Tiedens 2006; Van Kleef and Côté 2007) moderates the effectiveness of emotional expressions. The negotiation setting in the present study was such that the epistemic motivation and information processing needs as well as the interdependence structure were equal for both negotiators. However, in naturalistic interactions, such as in the present study, an asymmetric interdependence structure or different information processing motivations and needs may have implicitly evolved. Similarly, the negative transformational sequence of goal-oriented emotions may have been interpreted as justified by some negotiators and not by others (cf., Van Kleef and Côté 2007). These moderators may have emerged naturally during the negotiation interaction and potentially have affected how negotiators enacted the turning points and ultimately whether they reached a settlement or not. Assessing how the interdependence structure, information processing needs, and perceived justifiability affects the initiation and conclusion of the departure in controlled experimental studies may provide important insights into how negotiators successfully enact turning points.

A crucial question emerging from the present results is why some negotiators chose to embrace the departure and responded in kind via a reciprocal sequence while other negotiators failed to seize this opportunity for creating value and direct the negotiation towards positive grounds. A preliminary answer to this question is provided by Griessmair and Druckman (2018). They show that negotiators are more likely to embrace the departure when the turning point offer is made salient and proposed within an integrative-cooperative communication context. The turning point in the present study marked a clear change based on economic criteria; however, not all negotiators may have identified the turning point offer as potential transition towards creating value. Making the turning point offer salient by explicitly stating the intent, content, and function of the turning point offer may be required to increase the likelihood that the counterpart reciprocates the turning point offer (Griessmair and Druckman 2018).

Similarly, settlement dyads may have established a more cooperative-integrative climate prior to the turning point. Therefore, the turning point offer fell on fertile ground and was more likely to be reciprocated by the counterpart (Griessmair and Druckman 2018). Negotiators often mismatch the counterpart’s offer and take advantage of concessionary and conciliatory behavior (Allen et al. 1990; Hüffmeier et al. 2014). That is, rather than responding in kind to the conciliatory turning point offer and direct the negotiation towards positive grounds, they react by claiming value. Druckman et al. (2009) and Druckman and Olekalns (2013a) have found that how negotiators react to precipitants is influenced by their motivational frames and interpretative filters. Mismatching, interpretative filters, and motivational frames may be closely linked in explaining why impasse dyads employ negative transformational sequences that disrupt the change, and settlement dyads engage in reciprocal sequences that reinforce the positive departure. In a climate characterized by high trust and cooperative orientation, the turning point offer is interpreted as a sign of coming closer and the counterpart responds in kind. In a negative negotiation climate, on the other hand, the effort to come closer at the turning point offer is interpreted as a sign of weakness that is subsequently exploited with aggressive value claiming (cf., Van Kleef et al. 2010). In fact, research suggests that turning points are more likely to direct the negotiation towards positive grounds in a conducive negotiation climate (Griessmair and Druckman 2018). Disentangling how interpretive filters and the climate established by negotiators work together to influence the decision about how to react to the turning point offer constitutes an interesting avenue for future research.

Finally, following the tradition of negotiation process analyses (e.g., Brett et al. 1998; Olekalns and Smith 2000; Olekalns et al. 1996; Weingart et al. 1999, 2004), we did not manipulate the variables of interest but conducted ex-post analyses of the negotiation interactions. While this allows gaining insights into the interaction dynamics as they unfold naturally (Druckman and Olekalns 2013b), it poses the problem of inferring causality and isolating mechanisms and dynamics responsible for a specific effect. Thus, other factors—such as the aforementioned interpretative filters, motivational frames, interdependence structure, or whether the negotiators established a cooperative or competitive context prior to the departure—may have contributed to how negotiators enacted the turning point and whether they have ultimately reached an agreement. Prior turning point research has employed both controlled experiments (Druckman and Olekalns 2013a; Druckman et al. 2009; Griessmair and Druckman 2018) and retrospective analyses of negotiators’ interactions (Druckman 1986, 2001; Druckman et al. 1991). Since the relative weaknesses and strengths of the two approaches are complementary, Druckman and Olekalns (2013b) point out that both are required to fully understand turning points in negotiations. Thus, investigating the role of social signals in enacting departures, particularly the effectiveness of mixed communication, in a controlled experimental setting may be a fruitful avenue for future research.

## Footnotes

1 Druckman’s conception of turning points is related to the concept of ripeness (Zartman 2000), however, the latter focuses only on the first stage of Druckman’s model, the precipitants (Druckman 2004). A more detailed discussion of differences and similarities can be found in Druckman (2004).

2 A more detailed description of the emotional dimensions and the underlying methodology can be found in Griessmair (2017).

3 The variables contract imbalance and joint utility have been entered individually in the model to avoid multicollinearity. When entered simultaneously, the coefficients are JU: *β* = 0.019, *p* = 0.385; CI: *β* = − 3.713, *p* = 0.051.

4 For calculating the conditional effects, the constituent variables PD and SD have been mean centered.
